# Draft Genome Sequence of *Mycobacterium tuberculosis* KT-0192, Isolated in South Korea

**DOI:** 10.1128/genomeA.01601-15

**Published:** 2016-01-21

**Authors:** Sanghyun Lee, Seung Jung Han, Taesoo Kwon, Won Gi Yoo, Mi-ran Yun, Jong Seok Lee, Dae-Won Kim

**Affiliations:** a Division of Biosafety Evaluation and Control, Korea National Institute of Health, Korea Centers for Disease Control and Prevention, Chungbuk, Republic of Korea; b School of Biological Sciences, Seoul National University, Seoul, Republic of Korea; c Department of Environmental Medical Biology, Chung-Ang University, Seoul, Republic of Korea; d Department of Microbiology and Institute for Immunology and Immunological Diseases, Brain Korea 21 Plus Project for the Medical Sciences, Yonsei University College of Medicine, Seoul, Republic of Korea; e International Tuberculosis Research Center, Masanhappo-gu, Changwon, Republic of Korea

## Abstract

We report the draft genome sequence of totally drug-resistant (XDR) *Mycobacterium tuberculosis* KT-0192. This sequence will provide new insights into the main cause and evolution of drug resistance in *M. tuberculosis* KT-0192.

## GENOME ANNOUNCEMENT

Over 95% of tuberculosis (TB)-related deaths occur in low- and middle-income countries, and 9 million people fell ill with TB leading to 1.5 million deaths in 2013 (World Health Organization, 2014) (1). In the Republic of Korea, approximately 2,400 people die annually among the ~40,000-TB patients, which is the highest death rate from infectious diseases in the country. In addition, resistance to anti-TB drugs is an increasing problem accompanying the rising number of TB patients. Here, we report the draft genomic sequence of *Mycobacterium tuberculosis* KT-0192, a strain isolated from a South Korean patient diagnosed with totally drug-resistant TB (XDR-TB).

Based on spoligotyping*, M. tuberculosis* KT-0192 belongs to the non-Beijing family MTB, which was not susceptible to first- and second-line anti-TB drugs. *M. tuberculosis* KT-0192 was isolated from the sputum of a patient with active pulmonary TB at Masan National Hospital (NMH) in South Korea.

Genomic DNA was isolated from *M. tuberculosis* KT-0192 grown in 7H9 broth (Difco Laboratories, Detroit, MI, USA) supplemented with 10% (vol/vol) oleic acid-albumin-dextrose-catalase (OADC, Becton Dickinson, Sparks, MD, USA) for 1 month at 37°C.

The paired-end sequencing library was prepared using the NexTera DNA sample prep kit (Illumina, San Diego, CA, USA). The insert size of the library was 500 bp, and Illumina MiSeq (Illumina, San Diego, CA, USA) was used as the sequencing platform. A total of 6,762,782 reads were obtained by whole-genome sequencing, and the fold coverage was 362.9. Finally, 113 contigs were assembled from all reads using CLC Genomic Workbench (CLCbio, version 7.5) (2). The number of *N*_50_ contigs was 115,212. The genome size of the KT-0192 strain is 4,391,077, with a G+C content of 65.6%. Subsequently, we estimated a total of 4,156 open reading frames (ORFs), 45 tRNAs, and 3 rRNAs using the prediction programs Glimmer (3), tRNAScan-SE (4), and RNAMMER (5), respectively.

A total of 2,888 genes could be assigned to clusters of orthologous group (COG) functional categories: 125 were classified as cell wall/membrane/envelope biogenesis-related genes, and 252 were classified as lipid transport and metabolism-related genes, which were the most abundant except for general function genes (13.06%). The reason for this abundance is considered to be related to the fact that *M. tuberculosis* has more than 100 outer membrane proteins and uses lipids to construct the outer membrane (6). We identified 2,643 single nucleotide variants (SNVs) and 237 indels with GATK (7) (version 3.2.2). Among the SNVs, 74.8% (1,978) are in ORFs and the rest are in intergenic regions. Of the indels, 50.7% (77/152) of the insertions and 76.5% (65/85) of the deletions are in ORFs, with the rest in intergenic regions.

### Nucleotide sequence accession number. 

This whole-genome shotgun project has been deposited in the DDBJ/EMBL/GenBank under the accession number JUFF00000000.
